# Toward Personalized Control of Human Gut Bacterial Communities

**DOI:** 10.1128/mSystems.00165-17

**Published:** 2018-03-20

**Authors:** Lawrence A. David

**Affiliations:** a Department of Molecular Genetics and Microbiology, Duke University, Durham, North Carolina, USA; b Center for Genomic and Computational Biology, Duke University, Durham, North Carolina, USA

**Keywords:** human microbiome, microbiome engineering, systems biology

## Abstract

A key challenge in microbiology will be developing tools for manipulating human gut bacterial communities. Our ability to predict and control the dynamics of these communities is now in its infancy. To manage human gut microbiota, I am developing methods in three research domains. First, I am refining *in vitro* tools to experimentally study gut microbes at high throughput and in controlled settings. Second, I am adapting “big data” techniques to overcome statistical challenges confronting microbiota modeling. Third, I am testing study designs that can streamline human testing of microbiota manipulations. Assembling these methods creates new challenges, including training scientists who can work across disciplines such as engineering, ecology, and medicine. Nevertheless, I envision that overcoming these obstacles will enable my group to construct platforms that can personalize microbiota treatments, particularly ones based on diet. More broadly, I anticipate that such platforms will have applications across fields such as agriculture, biotechnology, and environmental management.

## Abstract (precis)

A key challenge in microbiology will be developing tools for manipulating human gut bacterial communities. Our ability to predict and control the dynamics of these communities is now in its infancy.

## PERSPECTIVE

Gut bacterial communities play outsized roles in human health and disease. To date, cross-sectional human cohort studies have associated specific enteric microbes with metabolic, immunological, and neurological illness, and interventional animal studies have identified causal roles for individual gut bacteria in a variety of diseases. Ongoing work focuses on establishing which bacteria cause disease in humans. A longer-term research goal is to prevent or ameliorate illness by changing levels of bacteria causally implicated in human health (1).

Currently, approaches for manipulating human gut microbiota are imprecise. Antibiotics can impact a wide range of commensal microbes and may inadvertently facilitate the growth of opportunistic pathogens (2). Fecal transplants deliver a panoply of bacteria to recipients (3), most of whose risks, benefits, and persistence are still incompletely understood. Multiple factors at present limit progress in designing specific microbiota manipulation techniques. Isolating gut bacteria is tedious, and gut microbial communities are hard to sample *in situ*. Modeling gut microbiota dynamics presents complex, highly multivariable statistical challenges. Translating animal microbiota findings into the clinic setting requires surmounting logistical barriers associated with human studies research. Overcoming these and other obstacles will be necessary for developing ways to reshape human gut microbiota in a controlled manner.

My strategy for tackling these challenges relies on method development. “New tools creat[e] new sciences,” observed the physicist Freeman Dyson (4). I focus on tools whose origins lie outside microbiological research; a technical breakthrough in one field often leads to advances in others. Indeed, human microbiome science itself serves as a case study in how borrowed methods can spur scientific revolutions: modern studies of microbially diverse gut populations exploded out of advances in DNA sequencing throughput stimulated by the Human Genome Project. I am now developing the following three sets of methods to facilitate human gut microbiota control.

1. I am working to refine *in vitro* methods for isolating and culturing human gut microbes. The volumes of these culture methods span 10 orders of magnitude. At the microscopic scale, I study bacteria using microfluidic tools developed by engineers over the past 3 decades. Microfluidics enables individual microbes to be isolated in wells or droplets that are only tens of microns in diameter. Microfluidic technology provides the ability to detect genes (5) and to measure traits across millions of individual cells in a single experiment (6)—throughput that exceeds traditional microbiological techniques by a thousand-fold. At the macroscopic scale, I analyze community-level interactions using the same continuous flow bioreactors as are found in the fields of industrial fermentation and wastewater treatment. These artificial gut models allow me to sidestep the challenges encountered in studying gut microbiota *in vivo*, which include constraints that limit sampling ability as well as those limiting measuring and manipulating the microbial environment (7). Together, my *in vitro* methods also let me investigate gut microbial biology independently of host processes such as an immune response or circadian rhythms.
2. I am also working to create *in silico* tools for modeling human gut microbiota. My efforts have benefited from “big data” analytical methods that have matured over the past 20 years. I am now applying machine learning and dimension reduction techniques to simplify microbiota data sets and to predict infection susceptibility in human cohorts. I am also incorporating mathematical tools from the geosciences to address statistical obstacles posed by the relative nature of most microbiota data sets. Colleagues and I merged these methods with phylogenetic models to design a data transform that preserves the integrity of statistical methods commonly run on microbiota data (8, 9). Lastly, I am now developing dynamic models that have their roots in commercial forecasting and engineering control systems from the 1960s (10). By adapting these models to microbiota data sets, we can infer microbiota therapy effects and predict new treatment outcomes.
3. Lastly, I am working to streamline experimental approaches for learning and validating the effects of gut microbiota therapies in humans. Translating basic science discoveries into the clinic setting can be impeded by the cost, length, and complexity of human studies research. Over the past years, my colleagues and I have explored alternative human study designs that rely on techniques such as self-tracking of healthy subjects, dense longitudinal sampling, and iOS device-enabled recording of lifestyle data. These approaches were inspired in part by human microbiota research methods that were published in the *Journal of the American Medical Association* (now *JAMA*) nearly a century ago (11). My resulting data showed that it is feasible to identify diet shifts that shape the gut microbiota in humans, using cohorts ranging in size from 2 to 10 volunteers and treatments that lasted for only days (12, 13). These findings show that research groups of even modest size can carry out interventional human cohort studies.

Of course, my strategy of developing tools across research domains presents challenges. Synergy is a primary one. In principle, I integrate methods in my research group using combined projects: dynamic models are applied to time-series harvested from our artificial gut; microfluidic assays are tested as diagnostics in human studies. But, in practice, achieving this integration demands that my laboratory members simultaneously work across disciplines such as engineering, ecology, medicine, microbiology, nutrition, probability, and statistics. Trainees doing interdisciplinary work confront the challenges of digesting disparate bodies of literature, debugging methods without relevant background coursework, and interpreting data while being potentially unaware of common sources of error. Nevertheless, a rich scientific payoff awaits. Locating scientists with complementary skills at the same time and in the same place provides an opportunity for immediate and unique intellectual connections, which should in turn lead to new insights into how we control the human gut microbiota.

Over the coming 5 years, I expect these insights, as well as other advances by colleagues, to translate into diagnostic tests and predictive dosing schemes that anticipate how gut microbiota will respond to treatments based on diet, probiotics, or drugs. As a proof of concept, I envision creating new microbiota treatments using typical diet components that gut microbiota ferment into crucial metabolic precursors and energy sources for colonic epithelial cells. Such dietary substances are often referred to as prebiotics. Notably, the effects of diet interventions often differ between individuals (14). I will therefore combine tools from my laboratory to identify bacterial taxa stimulated by prebiotics, to understand how prebiotic metabolism is shaped by overall diet, and to design optimal prebiotic intake strategies. Ultimately, I anticipate building a platform to personalize prebiotic treatments to individuals based on their gut microbiota and lifestyle. More broadly, I expect such a platform to have applications for bacterial communities outside the human gut. For example, these methods could be used to reshape microbiota that harvest nutrients for plants, synthesize chemicals through fermentation, and break down toxins in wastewater. Breakthroughs in treating human gut microbiota should thus ultimately propel microbial research in agriculture, biotechnology, and environmental management.
